# The association of cardiac troponin and cardiovascular events in patients with concomitant heart failure preserved ejection fraction and atrial fibrillation

**DOI:** 10.1186/s12872-023-03302-y

**Published:** 2023-05-24

**Authors:** Bum Sung Kim, Chang Hee Kwon, Haseong Chang, Ji-Hoon Choi, Hyun-Joong Kim, Sung Hea Kim

**Affiliations:** Division of Cardiology, Department of Medicine, Konkuk University Medical Center, 120-1 Neungdong-ro, Hwayang-dong, Gwangjin-gu, Seoul, 05030 Republic of Korea

**Keywords:** Atrial fibrillation, Heart failure with preserved ejection fraction, High-sensitivity cardiac troponin I, Cardiovascular event

## Abstract

**Background:**

Limited data are available for risk stratification in patients with atrial fibrillation (AF) and combined heart failure with preserved ejection fraction (HFpEF). We aimed to explore the prognostic utility of high-sensitivity cardiac troponin I (hs-cTnI) in patients with newly detected AF and concomitant HFpEF.

**Methods:**

From August 2014 to December 2016, 2,361 patients with newly detected AF were polled in a retrospective single-center registry. Of which, 634 patients were eligible for HFpEF diagnosis (HFA-PEFF score ≥ 5) and 165 patients were excluded with exclusion criteria. Finally, 469 patients are classified into elevated or non-elevated hs-cTnI groups based on the 99th percentile upper reference limit (URL). The primary outcome was the incidence of major adverse cardiac and cerebrovascular events (MACCE) during follow-up.

**Results:**

In 469 patients, 295 were stratified into the non-elevated hs-cTnI group (< 99th percentile URL of hs-cTnI) and 174 were placed in the elevated hs-cTnI group (≥ 99th percentile URL of hs-cTnI). The median follow-up period was 24.2 (interquartile range, 7.5–38.6) months. During the follow-up period, 106 patients (22.6%) in the study population experienced MACCE. In a multivariable Cox regression model, the elevated hs-cTnI group had a higher incidence of MACCE (adjusted hazard ratio [HR], 1.54; 95% confidence interval [CI], 1.08–2.55; *p* = 0.03) and coronary revascularization-caused readmission (adjusted HR, 3.86; 95% CI, 1.39–15.09; *p* = 0.02) compared with the non-elevated hs-cTnI group. The incidence of heart failure-caused readmission tended to occur more frequently in the elevated hs-cTnI group (8.5% versus 15.5%; adjusted HR, 1.52; 95% CI, 0.86–2.67; *p* = 0.08).

**Conclusions:**

One-fifth of patients with AF and concomitant HFpEF experienced MACCE during follow-up, and elevated hs-cTnI was independently associated with higher risk of MACCE, as driven by heart failure and revascularization-caused readmission. This finding suggested that hs-cTnI may be a useful tool in individualized risk stratification of future cardiovascular events in patients with AF and concomitant HFpEF.

**Supplementary Information:**

The online version contains supplementary material available at 10.1186/s12872-023-03302-y.

## Background

Heart failure with preserved ejection fraction (HFpEF) accounts for more than half of all hospital admissions for heart failure (HF), and concomitant atrial fibrillation (AF) is frequently observed [1]. The proportion of HFpEF patients with coexisting AF has been reported to range from 15% to as high as 65% in older populations [2–4]. The disorders share many common clinical features and are inextricably linked to each other [5]. AF is one of the precedents and predictors of HFpEF, and the advent of AF changes the clinical course of HFpEF by posing a higher risk of associated complications, including thromboembolic events, heart failure exacerbation, and an increase in mortality. Therefore, although early risk stratification and integrated care in patients with AF and concomitant HFpEF are important, these are challenging. Several studies have demonstrated a consistent association between elevated cardiac troponin level and risk of adverse cardiovascular outcomes among patients with AF and concomitant HF with reduced ejection fraction (HFrEF), even in the absence of chest pain or myocardial infarction [6–8]. In patients with HFpEF, cardiac troponin is frequently detectable, and higher level of high-sensitive cardiac troponin (hs-cTn) is associated with risk of adverse cardiovascular outcomes [9–11]. However, in patients with AF and concomitant HFpEF, the prognostic role of high-sensitivity cardiac troponin has not been established. Therefore, we aimed to explore the prognosis in patients with newly detected AF and concomitant HFpEF and investigate the prognostic utility of hs-cTn for clinical outcomes in patients with both disorders.

## Methods

### Study population

This study was performed using data from a single center registry of 2,361 patients with newly detected AF between August 2014 and December 2016 in Kon-Kuk Medical Center, Seoul, Republic of Korea. The electronic healthcare records of eligible patients were collected from this registry following the inclusion and exclusion criteria. The inclusion criteria were as follows: (1) high-sensitive cardiac troponin I (hs-cTnI) evaluated at the time of AF detection in an outpatient department, inpatient department, or emergency department; (2) HFpEF with symptoms and signs of HF; left ventricular ejection fraction (LVEF) ≥ 50%; and Heart Failure Association Pre-test assessment, echocardiography & natriuretic peptide, functional testing, and final etiology (HFA-PEFF) score ≥ 5; and (3) 18 years of age or older. The HFA-PEFF score, suggested by the Heart Failure Association of the European Society of Cardiology, is a stepwise approach for HFpEF diagnosis [12]. The score incorporates three domains—functional, morphological, and biomarker—to estimate the likelihood of HFpEF, and patients with more than 5 points are considered to have high probability for HFpEF. In patients with AF, separate criteria are applied regarding left atrium size and natriuretic peptide level to avoid overdiagnosis of HFpEF in AF. Table 1 demonstrates the major criteria (2 points) and minor criteria (1 point) of each domain used in this study. The exclusion criteria were (1) percutaneous coronary intervention (PCI) or coronary artery bypass grafting (CABG) surgery during index hospitalization; (2) estimated glomerular filtration rate (eGFR) less than 30 mL/min/1.73 m2 (using the Modified Diet in Renal Disease equation) at initial presentation and (3) insufficient clinical/laboratory data on the initial evaluation and follow-up visit.

**Table 1:** HFA-PEFF score for HFpEF diagnosis in patients with AF

	Functional	Morphologic (AF)	Biomarker (AF)
Major (2 points)	Septal e’ < 7 cm/s	LAVI > 40 ml/m2	BNP > 240 pg/ml
	E/e’ ≥ 15		
	PASP > 35 mmHg		
Minor (1 point)	E/e’ ratio 9—14	LAVI 34—40 ml/m2	BNP 105–240 pg/ml
≥ 5 points: High probability for HFpEF diagnosis			

*HFA-PEEF* Heart Failure Association Pre-test assessment, Echocardiography & natriuretic peptide, Functional testing, Final etiology, *HFpEF* Heart failure with preserved ejection fraction, *AF* Atrial fibrillation, *LAVI* Left atrium volume index, *BNP* Brain natriuretic peptide, *PASP* Pulmonary artery systolic pressure

A total of 469 patients were included in the final analysis. This observational study had no influence on patient treatment due to its retrospective design, and therapies were always provided at the discretion of the attending physicians. The investigation conforms with the principles outlined in the *Declaration of Helsinki. T*he Institutional Review Board of Kon-Kuk Medical Center approved the study protocol (KUH1010848) and waived the requirement for informed consent.

### Data collection and high-sensitivity cardiac troponin I assay

All patients underwent a complete baseline history survey, physical examination, 12-lead electrocardiogram (ECG), and laboratory exam on admission or upon the second visit to the outpatient department. The details of this registry have been published previously [13]. Cardiac troponin-I was assessed using the ARCHITECT STAT High-Sensitivity Troponin-I immunoassay on an ARCHITECT i2000SR immunoassay analyzer (Abbott Diagnostics, IL). The limit of detection was 1.9 ng/L. The 99th percentile upper reference limit (URL) was defined as 20.7 ng/L for men and 16.1 ng/L for women. The study population was categorized into two groups according to the 99th percentile URL of hs-cTnI level; patients below the 99th percentile URL were allocated to the non-elevated hs-cTnI group, and patients at/above the 99th percentile URL in placed in the elevated hs-cTnI group. Echocardiographic profiles were measured at an echocardiographic laboratory (Konkuk University Medical Center, Seoul, Korea) according to a protocol established by the American Society of Echocardiography. Clinical, laboratory, and outcome data were collected by a trained study coordinator using a standardized case report form and protocol.

### Study outcomes and definition

The primary outcome was major adverse cardiac and cerebrovascular events (MACCE), a composite of all-cause death, readmission caused by HF, and coronary revascularization or stroke during follow-up. The secondary outcomes were all-cause death, readmission caused by HF and coronary revascularization, and stroke during follow-up. HF causing readmission was defined as readmission with a primary diagnosis of HF on the basis of major and minor clinical criteria described by the Framingham Heart Study [14]. Coronary revascularization causing readmission was defined as readmission with any PCI or CABG surgery during follow-up. Stroke causing readmission was defined as readmission with a primary diagnosis of cerebral infraction with rapid-onset focal neurologic symptoms lasting at least 24 h. Mitral valve disease was defined as more than moderate degree of mitral stenosis or mitral insufficiency. H_2_FPEF score was constituted (1) a body mass index (BMI) > 30 kg/m2 (H); (2) use of ≥ 2 anti-hypertensive medications (H); (3) the presence of atrial fibrillation (F); (4) pulmonary hypertension defined as pulmonary artery systolic pressure > 35 mm Hg (P); (5) elderly with an age > 60 years (E); and (6) elevated filling pressures evident from E/eʹ > 9 (F). The presence of atrial fibrillation yields 3 points, a BMI > 30 kg/m2 yields 2 points, and all other variables yield 1 point [15].

### Statistical analysis

Baseline characteristics were summarized as mean ± standard deviation (SD) or median with interquartile range between elevated hs-cTnI and non-elevated hs-cTnI group. Continuous variables were compared using Student’s t test or Wilcoxon rank-sum test when applicable. Categorical data were analyzed using the Chi-square test. For clinical outcomes and the extended composite, the hazard ratio (HR) with 95% confidence interval and *p*-value were calculated using a Cox proportional hazard model with adjustment for covariates of age, female sex, previous HF, and use of loop diuretics. The cumulative event rates were estimated by the Kaplan–Meier method and were compared using log-rank tests. Multivariable Cox proportional hazard regression was performed to determine independent risk factors of MACCE during follow-up using variables that were significant (*p* < 0.10) in the univariate model. Statistical analyses were performed with SPSS version 20.0 (IBM, SPSS, Chicago, IL, USA). All tests were 2-tailed, and *p* < 0.05 was considered statistically significant.

## Results

### Baseline characteristics

**Fig. 1:**
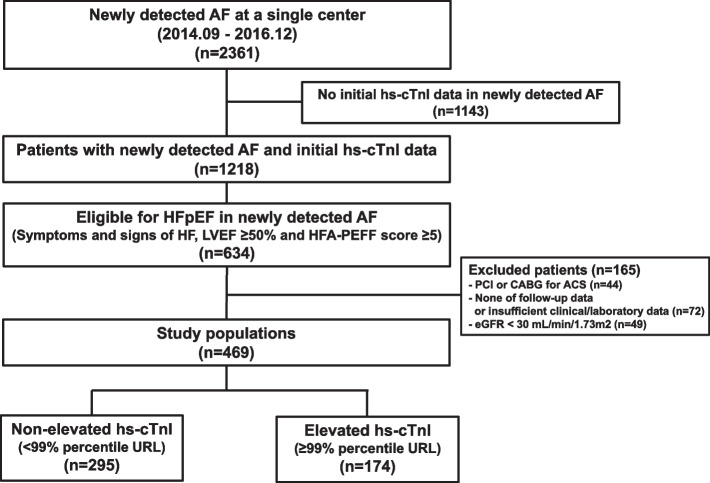
Schema of the study population distribution in the registry. hs-cTnI = high-sensitivity cardiac troponin I, AF = atrial fibrillation, HF = heart failure, LVEF = left ventricular ejection fraction, PCI = percutaneous coronary intervention, CABG = coronary artery bypass graft, ACS = acute coronary syndrome, URL = upper reference limit

Of the 2,361 patients with AF in the single-center registry, 1,218 underwent hs-cTnI evaluation at the time of presentation. Of these, 634 patients presented symptoms or signs of HF, LVEF ≥ 50%, and more than 5 points on HFA-PEFF. After applying the exclusion criteria, 165 patients were excluded. Finally, 469 patients with AF and concomitant HFpEF were selected for analysis; 174 patients (37.1%) were stratified into the elevated hs-cTnI group (≥ 99th percentile URL), and 295 patients (62.9%) were placed in the non-elevated hs-cTnI group (< 99th percentile URL) (Fig. 1). The median value of hs-cTnI was 10.4 ng/L (25th percentile, 3.8 ng/L; 75th percentile, 21.3 ng/L) in the overall population, 5.0 ng/L (25th percentile, 2.6 ng/L; 75th percentile, 9.4 ng/L) in the non-elevated hs-cTnI group, and 32.8 ng/L (25th percentile, 20.0 ng/L; 75th percentile, 217.4 ng/L) in the elevated hs-cTnI group. Clinical characteristics of patients and measures of cardiac structure and function according to hs-cTnI are summarized in Table 2. Compared with the non-elevated hs-cTnI group, those in the elevated hs-cTnI group tended to have old age, female sex, higher CHA2DS2-VASc score, higher BNP and creatinine level, higher prevalence of previous HF. Regarding echocardiographic parameters, the elevated hs-cTnI group had greater left atrium volume index and pulmonary artery systolic pressure, but septal e’ is lower in the elevated hs-cTnI group. In addition, patients in the elevated hs-cTnI group were more likely to receive loop diuretic medications.

**Table 2:** Baseline characteristics of the study population according to the 99th percentile upper reference limit of hs-cTnI

	Overall population	Non-elevation hs-cTnI (< 99th percentile URL)	Elevation hs-cTnI (≥ 99th percentile URL)	*P*-value
	(*n* = 469)	(*n* = 295)	(*n* = 174)	
Age (years)	72.4 ± 11.5	70.4 ± 11.9	75.7 ± 10.1	< 0.01
Age ≥ 70	298 (63.5)	170 (57.6)	128 (73.6)	< 0.01
Female	239 (51.0)	131 (44.4)	108 (62.1)	< 0.01
BMI (kg/m^2^)	28.7 ± 6.5	29.1 ± 5.7	27.8 ± 7.5	0.06
BMI ≥ 25	161 (34.3)	95 (32.2)	66 (37.9)	0.21
Systolic blood pressure (mmHg)	132.4 ± 25.7	131.9 ± 22.8	133.1 ± 30.0	0.66
Diastolic blood pressure (mmHg)	78.2 ± 18.1	79.5 ± 16.6	76.0 ± 15.1	0.34
Heart rate (rate/min)	85.0 ± 26.5	83.9 ± 26.3	86.5 ± 27.5	0.31
**Medical History**				
Hypertension	286 (61.0)	183 (62.0)	103 (59.2)	0.54
Diabetes mellitus	147 (31.3)	97 (32.9)	50 (28.7)	0.35
Current smoking	52 (11.1)	33 (11.2)	19 (10.9)	0.93
Dyslipidemia	54 (11.5)	39 (13.2)	15 (8.6)	0.13
Mitral valve disease	29 (6.2)	16 (5.4)	13 (7.5)	0.37
Previous PCI	24 (5.1)	16 (5.4)	8 (4.6)	0.69
Previous CABG	7 (1.5)	3 (1.0)	4 (2.3)	0.27
Previous heart failure	56 (11.9)	27 (9.2)	29 (16.7)	0.02
Previous stroke	103 (22.0)	56 (19.0)	47 (27.0)	0.05
**CHA2DS2-VASc**	3.3 ± 1.7	3.1 ± 1.7	3.7 ± 1.6	< 0.01
**CHA2DS2-VASc ≥ 3**	319 (68.0)	185 (62.7)	134 (77.0)	< 0.01
**AF detection at**				
Outpatient department	58 (12.4)	37 (12.5)	21 (12.1)	0.85
Inpatients department	101 (21.5)	69 (23.4)	32 (18.4)	0.21
Emergency department	310 (66.1)	189 (64.1)	122 (69.5)	0.08
**Laboratory parameter**				
Hemoglobin (g/dL)	12.8 ± 2.1	13.1 ± 2.1	12.4 ± 2.1	0.53
Creatinine (mg/dL)	1.0 ± 0.3	0.9 ± 0.2	1.1 ± 0.3	< 0.01
BNP (pg/dL)	455.5 ± 166.2	402.8 ± 165.5	546.2 ± 145.9	< 0.01
**Echocardiographic parameter**				
LVEF (%)	64.2 ± 6.6	64.8 ± 6.3	63.1 ± 7.1	0.06
Septal e’	6.2 ± 1.2	6.4 ± 1.2	6.1 ± 1.3	< 0.01
E/e’	13.9 ± 5.5	13.6 ± 5.1	14.5 ± 6.0	0.09
LAVI (mL/m^2^)	48.4 ± 5.1	48.5 ± 4.7	48.6 ± 6.0	0.03
LAVI > 40 mL/m^2^	448 (95.5)	282 (95.6)	166 (95.4)	0.92
PASP (mmHg)	33.7 ± 10.6	32.6 ± 9.1	36.0 ± 12.8	< 0.01
**H2FPEF score**	6.2 ± 1.2	6.2 ± 1.2	6.3 ± 1.1	0.08
**H2FPEF score ≥ 6points**	343 (73.1)	209 (70.8)	134 (77.0)	0.15
**Medication**				
Loop diuretics	160 (34.1)	86 (29.2)	74 (42.5)	< 0.01
Spironolactone	52 (11.1)	28 (9.5)	24 (13.8)	0.15
ACE-I/ARB	151 (32.2)	94 (31.9)	57 (32.8)	0.84
Beta-blocker	125 (26.7)	82 (27.8)	43 (24.7)	0.46
Anti-arrhythmic medication	58 (12.4)	40 (13.6)	18 (10.3)	0.31
Anti-coagulation	201 (42.9)	125 (42.4)	76 (43.7)	0.78
Anti-platelet agent	135 (28.8)	88 (29.8)	47 (27.0)	0.51

Values are mean ± standard deviation or n (%)

*hs-cTnI* high-sensitivity cardiac troponin I, *BMI* Body mass index, *URL* Upper reference limit, *PCI* Percutaneous coronary intervention, *CABG* Coronary artery bypass graft, *LVEF* Left ventricular ejection fraction, *LAVI* Left atrium volume index, *BNP* Brain natriuretic peptide, *PASP* Pulmonary artery systolic pressure, *H2FPEF score* Heavy, hypertensive, atrial fibrillation, pulmonary hypertension, elder, and filling pressure score, *ACE-I/ARB* Angiotensin-converting enzyme inhibitor/angiotensin receptor blocker

### Clinical outcomes according to high-sensitive cardiac troponin I

**Fig. 2:**
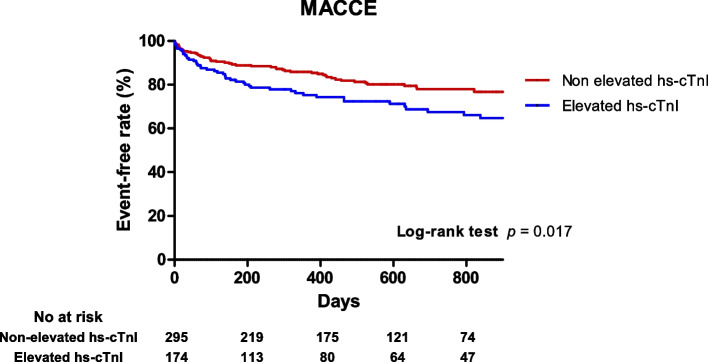
Kaplan–Meier curve of major adverse cardiac and cerebrovascular events between non-elevated hs-cTnI (< 99th percentile URL) and elevated hs-cTnI groups (≥ 99th percentile URL). MACCE = major adverse cardiac and cerebrovascular events; hs-cTnI = high-sensitivity cardiac troponin I, URL = upper reference limit

During a mean follow-up period of 24.2 months (interquartile range 7.5 to 38.6), 106 patients (22.6%) in the study population experienced MACCE, including 24 (5.1%) all-cause deaths, 52 (11.1%) HF-caused readmissions, 9 (1.9%) revascularization-caused readmissions, and 35 (7.4%) stroke-caused readmissions. Among patients with MACCE, 14 experienced multiple secondary outcomes. Table 3 demonstrates the clinical outcomes of the study population and compares unadjusted and adjusted hazard ratios between elevated and non-elevated hs-cTnI groups. Upon Cox regression analysis, compared with the non-elevated hs-cTnI group, the elevated hs-cTnI group demonstrated higher risk of MACCE (adjusted HR, 1.54; 95% confidence interval [CI], 1.08–2.55; *p* = 0.03) and revascularization-caused readmission (adjusted HR, 3.86; 95% CI, 1.39–15.09; *p* = 0.02). The incidence of HF-caused readmission tended to occur more frequently in the elevated hs-cTnI group (8.5% versus 15.5%; adjusted HR, 1.52; 95% CI, 0.86–2.67; *p* = 0.08). There were no significant differences in risk of all-cause death and stroke-caused readmission between elevated and non-elevated hs-cTnI groups. Figure 2 shows a Kaplan–Meier curve depicting the hazard for MACCE between the two groups.

**Table 3:** Clinical outcomes according to the 99th percentile upper reference limit of hs-cTnI in patients with newly detected AF and concomitant HFpEF

	Non-elevated hs-cTnI	Elevated hs-cTnI	Unadjusted HR	*P*-value	Adjusted HR^a^	*P*-value
	(< 99th percentile URL)	(≥ 99th percentile URL)				
	(*n* = 295)	(*n* = 174)	(95% CI)		(95% CI)	
**MACCE**	56 (19.0)	50 (28.7)	1.52 (1.03–2.24)	0.03	1.54 (1.08–2.55)	0.03
**All cause death**	12 (4.1)	12 (6.9)	1.51 (0.66–3.47)	0.32	1.34 (0.57–3.12)	0.51
**Heart failure-caused readmission**	25 (8.5)	27 (15.5)	1.88 (1.08–3.25)	0.02	1.52 (0.86–2.67)	0.08
**Revascularization-caused readmission**	3 (1.0)	6 (3.4)	3.58 (0.89–14.33)	0.07	3.86 (1.39–15.09)	0.02
**Stroke-caused readmission**	20 (6.8)	15 (8.6)	1.12 (0.56–2.24)	0.75	1.02 (0.49–2.08)	0.96

*MACCE* Major adverse cardiac and cerebrovascular events, *HFpEF* Heart failure with preserved ejection fraction, *AF* Atrial fibrillation, *hs-cTnI* high-sensitivity cardiac troponin I, *URL* Upper reference limit, *HR* Hazard ratio, *CI* Confidence interval

^a^ Adjusted factors: age, female sex, previous heart failure, and use of loop diuretics

### Independent predictor of clinical outcome

Crude associations between clinical variables and MACCE were determined using a Cox regression analysis model. Variables associated with MACCE in univariate analysis were then entered into a multivariable Cox regression analysis model in a step-wise fashion and retained in the model if *p* < 0.10. The independent predictors of MACCE in patients with newly detected AF and concomitant HFpEF were older age and elevated hs-cTnI group (Table 4). In the subgroup analysis, there was a nominally significant interaction in patients < 70 years (HR, 1.03; 95% CI, 0.40–2.64) or ≥ 70 years (HR, 1.66; 95% CI, 1.05–2.61; P for interaction = 0.03). In other subgroups, consistent association was observed between elevated hs-cTnI and risk of MACCE (Supplement figure).

**Table 4:** Independent predictor of major adverse cardiac and cerebrovascular event in patients with AF combined HFpEF

	Unadjusted		Adjusted^a^	
	Hazard ratio (95% CI)	*P*-value	Hazard ratio (95% CI)	*P*-value
Age (per 1 year old)	1.04 (1.02–1.05)	< 0.01	1.03 (1.01–1.05)	0.01
Female	1.05 (0.72–1.54)	0.77	.	.
Hypertension	1.11 (0.75–1.65)	0.59	.	.
Diabetes mellitus	1.06 (0.71–1.58)	0.77	.	.
BMI ≥ 25	1.67 (1.02–2.75)	0.04	1.18 (0.71–1.98)	0.51
Previous heart failure	1.58 (1.11–2.47)	0.01	1.25 (0.84–1.88)	0.26
Previous stroke	1.10 (0.69–1.76)	0.66	.	.
Use of loop diuretics	1.39 (0.86–2.51)	0.08	1.22 (0.73–2.04)	0.43
Elevated hs-cTnI (≥ 99th percentile URL)	1.69 (1.10–2.61)	0.01	1.59 (1.03–2.47)	0.03

*AF* Atrial fibrillation, *HFpEF* Heart failure preserved ejection fraction, *CI* Confidence interval, *BMI* Body mass index, *hs-cTnI* high-sensitivity cardiac troponin I, *URL* Upper reference limit

^a^ Adjusted covariate included age, BMI ≥ 25, previous heart failure, use of loop diuretics and elevated hs-cTnI (≥ 99th percentile URL)

## Discussion

In the present study, we explored the prognosis of patients with newly detected AF and concomitant HFpEF and investigated the association between hs-cTnI elevation and clinical outcomes. The results of this study can be summarized as follows. 1) In patients with newly detected AF and concomitant HFpEF, one-fifth experienced MACCE during a median of 24 months of follow-up; 2) The elevated hs-cTnI group had a higher risk of MACCE, as driven by heart failure and revascularization-caused readmission; and 3) elevated hs-cTnI group and older age were significant predictors of MACCE in patients with newly detected AF and concomitant HFpEF.

HFpEF and AF are frequently coexisting and interlinked clinical conditions. Their coexistence is a known negative prognostic factor [16]. In the Treatment of Preserved Cardiac Function Heart Failure with an Aldosterone Antagonist (TOPCAT) trial, 43% of patients with HFpEF had a history of AF or AF at enrollment. In patients with AF at enrollment, the primary composite outcome rate was 13.3 per 100 patient-years, and AF at enrollment was associated with an increased composite outcome of cardiovascular mortality, aborted cardiac arrest, or HF hospitalization [17]. In a more recent study regarding HFpEF, the Prospective Comparison of angiotensin receptor–neprilysin inhibitor with angiotensin-receptor blockers Global Outcomes in HF with Preserved Ejection Fraction (PARAGON-HF) trial, 54% of patients with HFpEF had both prior AF and AF at enrollment. In patients with AF at enrollment, the primary composite outcome rate was 15.3 per 100 patient-years, and AF at enrollment was associated with higher risk of total HF hospitalization and cardiovascular death [18]. In this study, we used a cohort of patients with newly detected AF and analyzed the clinical outcomes of those with combined HFpEF diagnosis. The incidence rate of MACCE was 22.6% during a median of 24.2 months of follow-up (11.3 per 100 patients-year), and this rate is comparable to previous studies.

In addition to the unfavorable prognosis of AF and concomitant HFpEF, there is no single medical treatment that shows survival benefits for such patients. Therefore, risk stratification is a preferential issue in the management of patients with AF-combined HFpEF [19]. However, there are limited data for risk stratification in patients with both disorders. In this study, patients with newly-detected AF and concomitant HFpEF were categorized into two groups according to the 99th percentile URL of hs-cTnI. The hs-cTnI elevation group had a higher risk of MACCE, as driven by heart failure and revascularization-caused readmission. In previous studies of patients with HFpEF, increased high-sensitivity cardiac troponin was associated with poor prognosis. In post-hoc analysis of the TOPCAT trial, higher hs-cTn level was independently associated with risk for cardiovascular death and HF hospitalization in patients with HFpEF [20]. In a large observational cohort study, abnormally elevated troponin level in patients with decompensated HFpEF was associated with higher risk of in-hospital and post-discharge adverse outcomes [11]. In population of HFpEF with specific etiology such as cardiac amyloidosis, cardiac troponin elevation frequently observed, even though potential interaction between etiology HFpEF and cardiac troponin release is uncertain. This disease entity had the greater refractoriness to treatment and higher mortality compared with other causes of HFpEF. The findings of our study suggest that measurement of high-sensitivity cardiac troponin may be an important tool for risk stratification, even in patients with HFpEF coexisting AF.

Diagnosis of HFpEF is often challenging due to its diverse phenotypes and is more complex in patients with AF and concomitant HFpEF. HF symptoms like dyspnea, fatigue, and impaired exercise tolerance are also the predominant symptoms of AF and largely overlap with HFpEF, complicating the definitive diagnosis of AF and concomitant HFpEF. Furthermore, in AF patients, common diastolic parameters are not readily applicable; it usually is accompanied by LA size enlargement and elevated natriuretic peptide levels. Recently, the Heart Failure Association of the European Society of Cardiology proposed the HFA-PEFF scoring system as a score-based algorithm to aid the diagnosis of HFpEF [12]. The HFA-PEFF scoring system has three domains—functional, morphological, and biomarker—to estimate the likelihood of HFpEF, and HFA-PEFF score ≥ 5 is diagnostic of HFpEF. In patients with AF, separate criteria are applied regarding left atrium size and natriuretic peptide level to avoid overdiagnosis of HFpEF in combined AF. In a previous study, high HFA-PEFF score (≥ 5 points) had a good correlation with the final diagnosis of HFpEF in well-phenotyped HFpEF cohorts [21]. In this study considering patients with newly detected AF, we used the HFA-PEFF score to categorize patients. Although advanced and etiology workup of HFpEF, as suggested by HFA consensus, was not performed, populations with a high probability for HFpEF (≥ 5 points on the HFA-PEFF) were selected among patients with newly detected AF. This approach reflected real clinical practice, with daily encounters of mixed clinical presentations of AF with combined symptoms and signs of HF and preserved LV systolic function. In this study, the predictors for MACCE in patients with newly detected AF and concomitant HFpEF were elevated hs-cTnI group and older age. In subgroup analysis, there was an interaction effect between older age and troponin elevation, indicating caution when assessing the risk of troponin elevation in elderly patients with AF and concomitant HFpEF.

There are several limitations to this study. First, this registry included newly detected AF patients, but not all patients in the registry were evaluated for hs-cTnI levels. In nearly half of patients, no initial hs-cTnI data were available and these patients were excluded from analysis. Therefore, selection bias associated with this factor is difficult to overcome. Second, clinical scenarios of AF detection were a mix with in-patient, out-patient and emergency department, although 87% patients of newly detected AF with concomitant HFpEF were originated from in-patient or emergency department visit. The clinical event and prognosis can be influenced by clinical scenarios of AF detection. Third, in the diagnostic HFA-PEEF algorithm, we could not use score variable of lateral e’, left ventricular mass index and relative wall thickness in HFA-PEEF score calculation, because of the retrospective nature of our registry. Therefore, it was not possible to identify the exact HFA-PEEF score calculated in this study. And, we did not perform etiology workup for HFpEF, such as an exercise stress test or cardiac magnetic resonance. Fourth, restoration of sinus rhythm might be an effective treatment option for patients with AF to reduce the burden of heart failure. However, we could not identify rhythm status of study population during follow up. Fifth, even though echocardiographic parameters were measured according to a protocol established by the American Society of Echocardiography, there was no external validation of echocardiographic data and no detailed information of underlying rhythm during echocardiographic measurement. Sixth, in this study, the rate of anti-coagulation therapy was lower than that of CHA2DS2-VASc score. Although the discrepancy between anti-coagulation use and anti-coagulation indication was presumed to stem from potential of bleeding risk or previous bleeding event, we did not have detailed information of previous bleeding event or frailty in study populations. Finally, recent guidelines recommended sodium-glucose cotransporter-2 inhibitor or angiotensin receptor-neprilysin inhibitors in management of HFpEF [22]. However, this registry was carried out before medications were introduced at this country, and our results did not reflect the effect of newly recommended medications.

## Conclusion

In patients with AF and concomitant HFpEF, one-fifth experienced MACCE during follow-up, and elevated hs-cTnI is independently associated with a higher risk of MACCE, as driven by heart failure and revascularization-caused readmission. Our findings suggest that hs-cTnI may aid the individualized risk stratification of future cardiovascular events in patients with AF and concomitant HFpEF.

## Supplementary Information

**Additional file 1: Supplement figure.** Comparative hazard ratio of major adverse cardiac and cerebrovascular events between non-elevated hs-cTnI and elevated hs-cTnI groups in each subgroup.

## Data Availability

The datasets used during this research are not publicly available because of privacy and ethical restrictions. However, they are available from the corresponding author on reasonable request.

## Abbreviations

HFpEF: Heart failure with preserved ejection fraction
HF: Heart failure
AF: Atrial fibrillation
HFrEF: Heart failure with reduced ejection fraction
hs-cTnI: High-sensitive cardiac troponin I
LVEF: Left ventricular ejection fraction
HFA-PEFF: Heart Failure Association Pre-test assessment, echocardiography & natriuretic peptide, functional testing, and final etiology
URL: Upper reference limit
CABG: Coronary artery bypass grafting
PCI: Percutaneous coronary intervention
eGFR: Estimated glomerular filtration rate
BMI: Body mass index
MACCE: Major adverse cardiac and cerebrovascular events
