# Supplementary material for: The association of cardiac troponin and cardiovascular events in patients with concomitant heart failure preserved ejection fraction and atrial fibrillation
Source: BMC Cardiovasc Disord. 2023 May 24;23:273. doi: 10.1186/s12872-023-03302-y (PMC10207698; doi:10.1186/s12872-023-03302-y)
Supplement: Supplementary file 1 — Additional file 1: Supplement figure. Comparative hazard ratio of major adverse cardiac and cerebrovascular events between non-elevated hs-cTnI and elevated hs-cTnI groups in each subgroup. [file 12872_2023_3302_MOESM1_ESM.docx]

**Supplement figure.** Comparative hazard ratio of major adverse cardiac and cerebrovascular events between non-elevated hs-cTnI (<99th percentile URL) and elevated hs-cTnI groups (≥99th percentile URL) in each subgroup


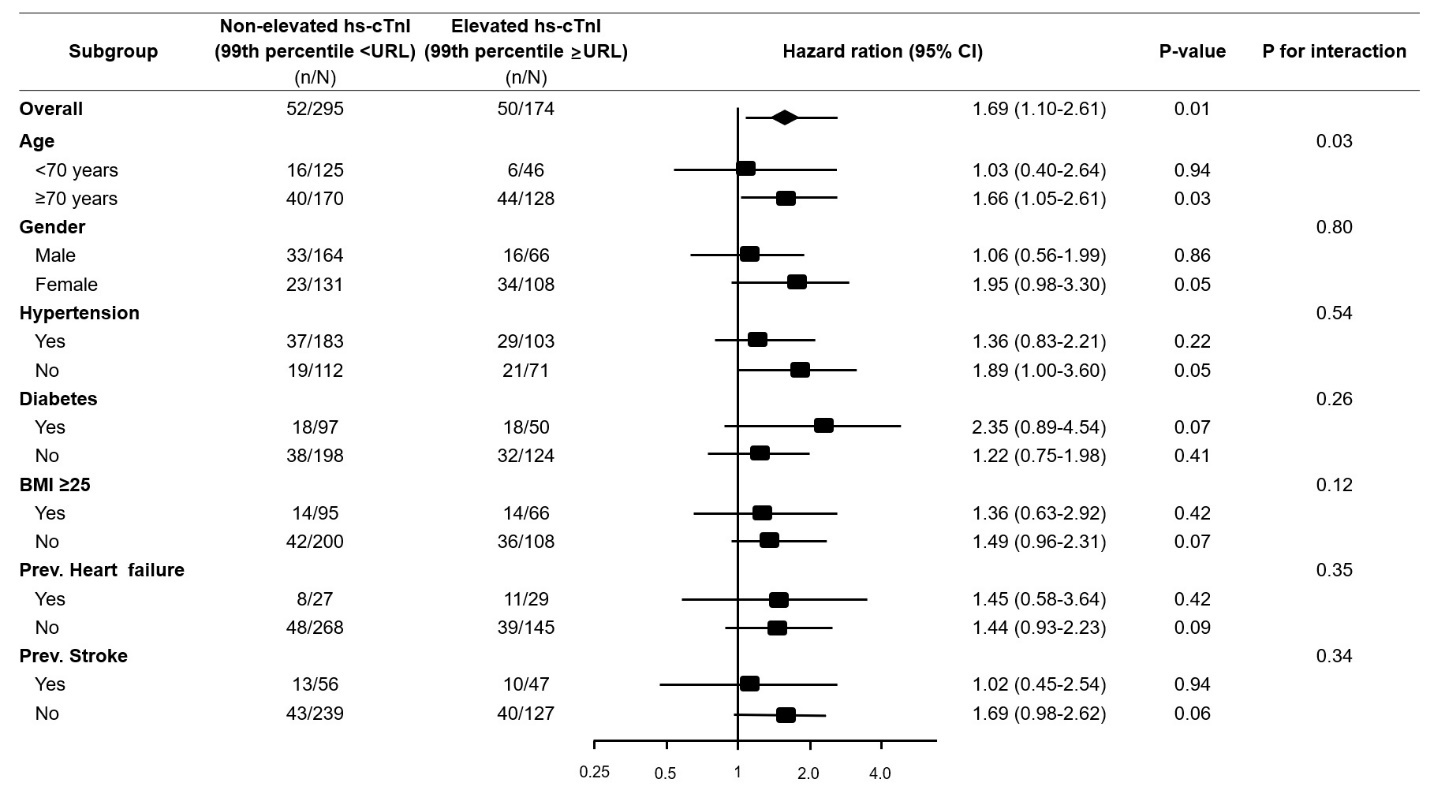


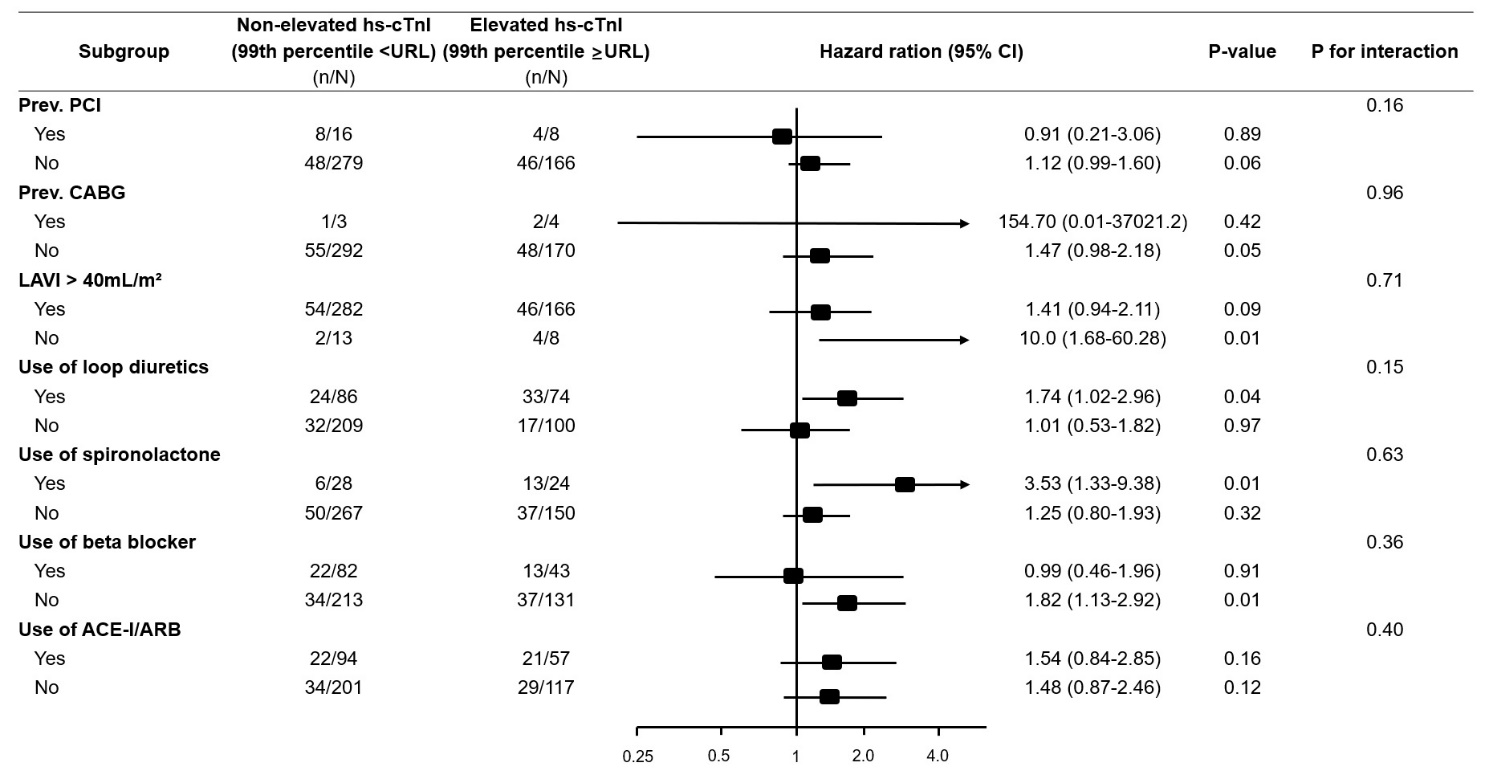


hs-TnI = high-sensitivity cardiac troponin I; BMI = body mass index; URL = upper reference limit; CI = confidence interval; PCI = percutaneous coronary intervention; CABG = coronary artery bypass graft; LAVI = left atrium volume index; ACE-I/ARB = angiotensin-converting enzyme inhibitor/angiotensin receptor blocker
